# Global data on earthworm abundance, biomass, diversity and corresponding environmental properties

**DOI:** 10.1038/s41597-021-00912-z

**Published:** 2021-05-21

**Authors:** Helen R. P. Phillips, Elizabeth M. Bach, Marie L. C. Bartz, Joanne M. Bennett, Rémy Beugnon, Maria J. I. Briones, George G. Brown, Olga Ferlian, Konstantin B. Gongalsky, Carlos A. Guerra, Birgitta König-Ries, Julia J. Krebs, Alberto Orgiazzi, Kelly S. Ramirez, David J. Russell, Benjamin Schwarz, Diana H. Wall, Ulrich Brose, Thibaud Decaëns, Patrick Lavelle, Michel Loreau, Jérôme Mathieu, Christian Mulder, Wim H. van der Putten, Matthias C. Rillig, Madhav P. Thakur, Franciska T. de Vries, David A. Wardle, Christian Ammer, Sabine Ammer, Miwa Arai, Fredrick O. Ayuke, Geoff H. Baker, Dilmar Baretta, Dietmar Barkusky, Robin Beauséjour, Jose C. Bedano, Klaus Birkhofer, Eric Blanchart, Bernd Blossey, Thomas Bolger, Robert L. Bradley, Michel Brossard, James C. Burtis, Yvan Capowiez, Timothy R. Cavagnaro, Amy Choi, Julia Clause, Daniel Cluzeau, Anja Coors, Felicity V. Crotty, Jasmine M. Crumsey, Andrea Dávalos, Darío J. Díaz Cosín, Annise M. Dobson, Anahí Domínguez, Andrés Esteban Duhour, Nick van Eekeren, Christoph Emmerling, Liliana B. Falco, Rosa Fernández, Steven J. Fonte, Carlos Fragoso, André L. C. Franco, Abegail Fusilero, Anna P. Geraskina, Shaieste Gholami, Grizelle González, Michael J. Gundale, Mónica Gutiérrez López, Branimir K. Hackenberger, Davorka K. Hackenberger, Luis M. Hernández, Jeff R. Hirth, Takuo Hishi, Andrew R. Holdsworth, Martin Holmstrup, Kristine N. Hopfensperger, Esperanza Huerta Lwanga, Veikko Huhta, Tunsisa T. Hurisso, Basil V. Iannone, Madalina Iordache, Ulrich Irmler, Mari Ivask, Juan B. Jesús, Jodi L. Johnson-Maynard, Monika Joschko, Nobuhiro Kaneko, Radoslava Kanianska, Aidan M. Keith, Maria L. Kernecker, Armand W. Koné, Yahya Kooch, Sanna T. Kukkonen, H. Lalthanzara, Daniel R. Lammel, Iurii M. Lebedev, Edith Le Cadre, Noa K. Lincoln, Danilo López-Hernández, Scott R. Loss, Raphael Marichal, Radim Matula, Yukio Minamiya, Jan Hendrik Moos, Gerardo Moreno, Alejandro Morón-Ríos, Hasegawa Motohiro, Bart Muys, Johan Neirynck, Lindsey Norgrove, Marta Novo, Visa Nuutinen, Victoria Nuzzo, P. Mujeeb Rahman, Johan Pansu, Shishir Paudel, Guénola Pérès, Lorenzo Pérez-Camacho, Jean-François Ponge, Jörg Prietzel, Irina B. Rapoport, Muhammad Imtiaz Rashid, Salvador Rebollo, Miguel Á. Rodríguez, Alexander M. Roth, Guillaume X. Rousseau, Anna Rozen, Ehsan Sayad, Loes van Schaik, Bryant Scharenbroch, Michael Schirrmann, Olaf Schmidt, Boris Schröder, Julia Seeber, Maxim P. Shashkov, Jaswinder Singh, Sandy M. Smith, Michael Steinwandter, Katalin Szlavecz, José Antonio Talavera, Dolores Trigo, Jiro Tsukamoto, Sheila Uribe-López, Anne W. de Valença, Iñigo Virto, Adrian A. Wackett, Matthew W. Warren, Emily R. Webster, Nathaniel H. Wehr, Joann K. Whalen, Michael B. Wironen, Volkmar Wolters, Pengfei Wu, Irina V. Zenkova, Weixin Zhang, Erin K. Cameron, Nico Eisenhauer

**Affiliations:** 1 German Centre for Integrative Biodiversity Research (iDiv) Halle-Jena-Leipzig, Puschstrasse 4, 04103 Leipzig, Germany; 2 Institute of Biology, Leipzig University, Puschstrasse 4, 04103 Leipzig, Germany; 3 Department of Environmental Science, Saint Mary’s University, Halifax, Nova Scotia Canada; 4 Global Soil Biodiversity Initiative and School of Global Environmental Sustainability, Colorado State University, Fort Collins, CO 80523 USA; 5 Department of Biology, Colorado State University, Fort Collins, CO 80523 USA; 6 Universidade Positivo, Rua Prof. Pedro Viriato Parigot de Souza, 5300, Curitiba, PR 81280-330 Brazil; 7 Center of Functional Ecology, Department of Life Sciences, University of Coimbra, Calçada Martins de Freitas, 3000-456 Coimbra, Portugal; 8 Institute of Biology, Martin Luther University Halle-Wittenberg, Am Kirchtor 1, 06108 Halle (Saale), Germany; 9 Centre for Applied Water Science, Institute for Applied Ecology, Faculty of Science and Technology, University of Canberra, Canberra, Australia; 10 Departamento de Ecología y Biología Animal, Universidad de Vigo, 36310 Vigo, Spain; 11 Embrapa Forestry, Estrada da Ribeira, km. 111, C.P. 231, Colombo, PR 83411-000 Brazil; 12 A.N. Severtsov Institute of Ecology and Evolution, Russian Academy of Sciences, Leninsky pr., 33 Moscow, 119071 Russia; 13 M.V. Lomonosov Moscow State University, Leninskie Gory, 1 Moscow, 119991 Russia; 14 Institute of Computer Science, Friedrich Schiller University Jena, Ernst-Abbe-Platz 2, 07743 Jena, Germany; 15 European Commission, Joint Research Centre (JRC), Ispra, Italy; 16 Department of Terrestrial Ecology, Netherlands Institute of Ecology (NIOO-KNAW), 6700 Wageningen, AB The Netherlands; 17 Senckenberg Museum for Natural History Görlitz, Department of Soil Zoology, 02826 Görlitz, Germany; 18 Biometry and Environmental System Analysis, University of Freiburg, Tennenbacher Str. 4, 79106 Freiburg, Germany; 19 Institute of Biodiversity, Friedrich Schiller University Jena, Dornburger-Str. 159, 07743 Jena, Germany; 20 CEFE, Univ Montpellier, CNRS, EPHE, IRD, Univ Paul Valéry Montpellier 3, Montpellier, France; 21 Sorbonne Université, Institut d’Ecologie et des Sciences de l’Environnement, 75005 Paris, France; 22 Centre for Biodiversity Theory and Modelling, Theoretical and Experimental Ecology Station, CNRS, 09200 Moulis, France; 23 Sorbonne Université, Institute of Ecology and Environmental Sciences of Paris (UMR 7618 IEES-Paris, CNRS, INRA, UPMC, IRD, UPEC), 4 place Jussieu, 75000 Paris, France; 24 INRA, IRD, Institut d’Ecologie et des Sciences de l’Environnement de Paris, F-75005 Paris, France; 25 Department of Biological, Geological and Environmental Sciences, University of Catania, Via Androne 81, 95124 Catania, Italy; 26 Laboratory of Nematology, Wageningen University, PO Box 8123, 6700 Wageningen, ES The Netherlands; 27 Institute of Biology, Freie Universität Berlin, 14195 Berlin, Germany; 28 Institute for Biodiversity and Ecosystem Dynamics, University of Amsterdam, Amsterdam, The Netherlands; 29 Asian School of the Environment, Nanyang Technological University, Singapore, 639798 Singapore; 30 Centre of Biodiversity and Sustainable Landuse, University of Göttingen, Büsgenweg 1, Göttingen, Germany; 31 Silviculture and Forest Ecology of the Temperate Zones, University of Göttingen, Büsgenweg 1, Göttingen, Germany; 32 Forest Sciences and Forest Ecology, University of Göttingen, Büsgenweg 1, Göttingen, Germany; 33 Institute for Agro-Environmental Sciences, National Agriculture and Food Research Organization, 3-1-3 Kan-nondai, Tsukuba, Ibaraki Japan; 34 Land Resource Management and Agricultural Technology, University of Nairobi, Kapenguria Road, Off Naivasha Road, P.O Box 29053, Nairobi, Kenya; 35 Rwanda Institute for Conservation Agriculture, KG 541, Kigali, Rwanda; 36 Health & Biosecurity, CSIRO, PO Box 1700, Canberra, Australia; 37 Department of Animal Science, Santa Catarina State University, Chapecó, SC 89815-630 Brazil; 38 Experimental Infrastructure Platform (EIP), Leibniz Centre for Agricultural Landscape Research, Eberswalder Str. 84, Müncheberg, Germany; 39 Départment de biologie, Université de Sherbrooke, Sherbrooke, Québec Canada; 40 Geology Department, FCEFQyN, ICBIA-CONICET (National Scientific and Technical Research Council), National University of Rio Cuarto, Ruta 36 Km, 601 Río Cuarto, Argentina; 41 Department of Ecology, Brandenburg University of Technology, Konrad-Wachsmann-Allee 6, Cottbus, Germany; 42 Eco&Sols, Univ Montpellier, IRD, INRAE, CIRAD, Institut Agro, Montpellier, France; 43 Natural Resources, Cornell University, Ithaca, NY USA; 44 Earth Institute, University College Dublin, Belfield, Dublin, 4 Ireland; 45 School of Biology and Environmental Science, University College Dublin, Belfield, Dublin, Ireland; 46 Department of Entomology, Cornell University, 3132 Comstock Hall, Ithaca, NY USA; 47 EMMAH, UMR 1114, INRA, Site Agroparc, Avignon, France; 48 The School of Agriculture, Food and Wine, The Waite Research Institute, The University of Adelaide, PMB 1 Glen Osmond, Australia; 49 Faculty of Forestry, University of Toronto, 33 Willcocks Street, Toronto, Canada; 50 Laboratoire Écologie et Biologie des Interactions, équipe EES, UMR CNRS 7267, Université de Poitiers, 5 rue Albert Turpain, Poitiers, France; 51 UMR ECOBIO (Ecosystems, Biodiversity, Evolution) CNRS-Université de Rennes, Station Biologique, 35380 Paimpont, France; 52 ECT Oekotoxikologie GmbH, Boettgerstr. 2-14, Floersheim, Germany; 53 Institute of Biological, Environmental and Rural Sciences, Aberystwyth Universtiy, Plas Gogerddan, Aberystwyth, SY24 3EE United Kingdom; 54 School for Agriculture, Food and the Environment, Royal Agricultural University, Stroud Road, Cirencester, GL7 6JS United Kingdom; 55 Odum School of Ecology, University of Georgia, 140 E Green Street, Athens, USA; 56 Department of Biological Sciencies, SUNY Cortland, 1215 Bowers Hall, Cortland, USA; 57 Biodiversity, Ecology and Evolution, Faculty of Biology, University Complutense of Madrid, José Antonio Novais, 12, Madrid, Spain; 58 Yale School of the Environment, Yale University, 370 Prospect St, New Haven, CT USA; 59 Departamento de Ciencias Básicas, Universidad Nacional de Luján, Argentina - INEDES (Universidad Nacional de Luján - CONICET), Luján, Argentina; 60 Louis Bolk Institute, Kosterijland 3-5, Bunnik, The Netherlands; 61 Department of Soil Science, University of Trier, Campus II, Behringstraße 21, Trier, Germany; 62 Departamento de Ciencias Básicas, Instituto de Ecología y Desarrollo Sustentable, Universidad Nacional de Luján, Av. Constitución y Ruta 5, Luján, Argentina; 63 Animal Biodiversity and Evolution, Institute of Evolutionary Biology, Passeig Marítim de la Barceloneta 37, Barcelona, Spain; 64 Department of Soil and Crop Sciences, Colorado State University, 1170 Campus Delivery, Fort Collins, CO USA; 65 Biodiversity and Systematic Network, Institute of Ecology A.C., El Haya, Xalapa, Veracruz, 91070 Mexico; 66 Department of Biology, Colorado State University, 200 West Lake Street, Fort Collins, CO USA; 67 Department of Biological Sciences and Environmental Studies, University of the Philippines Mindanao, Tugbok District, Davao Philippines; 68 Laboratory of Environmental Toxicology and Aquatic Ecology, Environmental Toxicology Unit - GhEnToxLab, Ghent University, Campus Coupure, Coupure Links 653, Ghent, Belgium; 69 Center for Forest Ecology and Productivity RAS, Profsoyuznaya st. 84/32 bldg. 14, Moscow, Russia; 70 Razi University, Kermanshah, Iran; 71 United States Department of Agriculture, Forest Service, International Institute of Tropical Forestry, 1201 Ceiba Street, San Juan, Puerto Rico; 72 Department of Forest Ecology and Management, Swedish University of Agricultural Sciences, Skogsmarksgrand 17, 901 83 Umeå, Sweden; 73 Department of Biology, University of Osijek, Cara Hadrijana 8 A, Osijek, Croatia; 74 Agriculture engineering, Agroecology Postgraduate Program, Maranhão State University, Avenida Lourenço Vieira da Silva 1000, São Luis, Brazil; 75 Department of Jobs, Precincts and Regions, Agriculture Victoria, Chiltern Valley Road, Rutherglen, Australia; 76 Faculty of Agriculture, Kyushu University, 394 Tsubakuro, Sasaguri, Fukuoka, 811-2415 Japan; 77 Minnesota Pollution Control Agency, 520 Lafayette Road, St Paul, MN USA; 78 Department of Bioscience, Aarhus University, Vejlsøvej 25, Aarhus, Denmark; 79 Department of Biological Science, Northern Kentucky University, 1 Nunn Drive, Highland Heights, KY USA; 80 Agricultura Sociedad y Ambiente, El Colegio de la Frontera Sur, Av. Polígono s/n Cd. Industrial Lerma, Campeche, Campeche, Mexico; 81 Soil Physics and Land Management Group, Wageningen University & Research, Droevendaalsteeg 4, Wageningen, The Netherlands; 82 Dept. of Biological and Environmental Sciences, University of Jyväskylä, Box 35, Jyväskylä, Finland; 83 College of Agriculture, Environmental and Human Sciences, Lincoln University of Missouri, Jefferson City, MO 65101 USA; 84 School of Forest Resources and Conservation, University of Florida, Gainesville, USA; 85 Sustainable Development and Environmental Engineering, University of Agricultural Sciences and Veterinary Medicine of Banat “King Michael the 1st of Romania” from Timisoara, Calea Aradului 119, Timisoara, Romania; 86 Institute for Ecosystem Research, University of Kiel, Olshausenstrasse 40, 24098 Kiel, Germany; 87 Tartu College, Tallinn University of Technology, Puiestee 78, Tartu, Estonia; 88 Department of Soil and Water Systems, University of Idaho, 875 Perimeter Drive MS, 2340 Moscow, USA; 89 Faculty of Food and Agricultural Sciences, Fukushima University, Kanayagawa 1, Fukushima, Japan; 90 Department of Environment, Faculty of Natural Sciences, Matej Bel University, Tajovského 40, Banská Bystrica, Slovakia; 91 UK Centre for Ecology & Hydrology, Library Avenue, Bailrigg, Lancaster, United Kingdom; 92 Land Use and Governance, Leibniz Centre for Agricultural Landscape Research, Eberswalder Str. 84, Müncheberg, Germany; 93 UFR Sciences de la Nature, UR Gestion Durable des Sols, Université Nangui Abrogoua, Abidjan, Côte d’Ivoire; 94 Faculty of Natural Resources and Marine Sciences, Tarbiat Modares University, 46417-76489 Noor, Mazandaran Iran; 95 Production Systems, Natural Resources Institute Finland, Survontie 9 A, Jyväskylä, Finland; 96 Department of Zoology, Pachhunga University College, Aizawl, Mizoram India; 97 Skolkovo Institute of Science and Technology, 30-1 Bolshoy Boulevard, Moscow, 121205 Russia; 98 SAS, INRAE, Institut Agro, 35042 Rennes, France; 99 Tropical Plant and Soil Sciences, College of Tropical Agriculture and Human Resources, University of Hawai’i at Manoa, 3190 Maile Way, St. John 102, Honolulu, USA; 100 Ecologia Aplicada, Instituto de Zoologia y Ecologia Tropical, Universidad Central de Venezuela, Los Chaguaramos, Ciudad Universitaria, Caracas, Venezuela; 101 Department of Natural Resource Ecology and Management, Oklahoma State University, 008C Ag Hall, Stillwater USA; 102 UPR Systèmes de Pérennes, CIRAD, Univ Montpellier, TA B-34/02 Avenue Agropolis, Montpellier, France; 103 Department of Forest Ecology, Faculty of Forestry and Wood Technology, Czech University of Life Sciences Prague, Kamýcká 129, Prague, Czech Republic; 104 Tochigi Prefectural Museum, 2-2 Mutsumi-cho, Utsunomiya, Japan; 105 Thuenen-Institute of Biodiversity, Bundesallee 65, Braunschweig, Germany; 106 Thuenen-Institute of Organic Farming, Trenthorst 32, Westerau, Germany; 107 Plant Biology, Ecology and Earth Science, INDEHESA, University of Extremadura, Plasencia, Spain; 108 Conservación de la Biodiversidad, El Colegio de la Frontera Sur, Av. Rancho, poligono 2 A, Cd. Industrial de Lerma, Campeche, Mexico; 109 Department of Environmental Systems Science, Faculty of Science and Engineering, Doshisha University, Kyoto, 602-8580 Japan; 110 Department of Earth & Environmental Sciences, Division of Forest, Nature and Landscape, KU Leuven, Celestijnenlaan 200E Box, 2411 Leuven, Belgium; 111 Research Institute for Nature and Forest, Gaverstraat 35, 9500 Geraardsbergen, Belgium; 112 School of Agricultural, Forest and Food Sciences, Bern University of Applied Sciences, Länggasse 85, Zollikofen, Switzerland; 113 Soil Ecosystems, Natural Resources Institute Finland (Luke), Tietotie 4, Jokioinen, Finland; 114 Natural Area Consultants, 1 West Hill School Road, Richford, NY USA; 115 Department of Zoology, PSMO College, Tirurangadi, Malappuram, Kerala, India, Malappuram, India; 116 CSIRO Ocean and Atmosphere, CSIRO, New Illawarra Road, Lucas Heights, NSW Australia; 117 UMR7144 Adaptation et Diversité en Milieu Marin, Station Biologique de Roscoff, CNRS/Sorbonne Université, Place Georges Teissier, Roscoff, France; 118 Phipps Conservatory and Botanical Gardens, Pittsburgh, PA 15213 USA; 119 UMR SAS, INRAE, Institut Agro Agrocampus Ouest, 35000 Rennes, France; 120 Forest Ecology and Restoration Group, Department of Life Sciences, University of Alcalá, 28805 Alcalá De Henares, Spain; 121 Adaptations du Vivant, CNRS UMR 7179, Muséum National d’Histoire Naturelle, 4 Avenue du Petit Château, Brunoy, France; 122 Department of Ecology and Ecosystem Management, Technical University of Munich, Emil-Ramann-Str. 2, 85354 Freising, Germany; 123 Tembotov Institute of Ecology of Mountain Territories, Russian Academy of Sciences, I. Armand, 37a, Nalchik, Russia; 124 Center of Excellence in Environmental Studies, King Abdulaziz University, P.O Box 80216, Jeddah, 21589 Saudi Arabia; 125 Global Change Ecology and Evolution Research Group (GloCEE), Department of Life Sciences, University of Alcalá, 28805 Alcalá De Henares, Spain; 126 Department of Forest Resources, University of Minnesota, 1530 Cleveland Ave. N, St. Paul, USA; 127 Friends of the Mississippi River, 101 E 5th St. Suite 2000, St Paul, USA; 128 Biology, Biodiversity and Conservation Postgraduate Program, Federal University of Maranhão, Avenida dos Portugueses 1966, São Luis, Brazil; 129 Institute of Environmental Sciences, Jagiellonian University, Gronostajowa 7, Kraków, Poland; 130 College of Natural Resources, University of Wisconsin, Stevens Point, WI 54481 USA; 131 The Morton Arboretum, 4100 Illinois Route 53, Lisle, IL 60532 USA; 132 Department Engineering for Crop Production, Leibniz Institute for Agricultural Engineering and Bioeconomy (ATB), Max-Eyth-Allee 100, Potsdam, Germany; 133 School of Agriculture and Food Science, University College Dublin, Agriculture and Food Science Centre, Dublin, Ireland; 134 UCD Earth Institute, University College Dublin, Dublin, Ireland; 135 Landscape Ecology and Environmental Systems Analysis, Institute of Geoecology, Technische Universität Braunschweig, Langer Kamp 19c, Braunschweig, Germany; 136 Department of Ecology, University of Innsbruck, Technikerstrasse 25, Innsbruck, Austria; 137 Institute for Alpine Environment, Eurac Research, Viale Druso 1, Bozen/Bolzano, Italy; 138 Laboratory of Ecosystem Modelling, Institute of Physicochemical and Biological Problems in Soil Science of the Russian Academy of Sciences, Institutskaya str., 2, Pushchino, Russia; 139 Laboratory of Computational Ecology, Institute of Mathematical Problems of Biology RAS – the Branch of Keldysh Institute of Applied Mathematics of the Russian Academy of Sciences, Vitkevicha str., 1, Pushchino, Russia; 140 Department of Zoology, Khalsa College Amritsar, Amritsar, Punjab India; 141 Department of Earth and Planetary Sciences, Johns Hopkins University, 3400 N. Charles Street, Baltimore, USA; 142 Department of animal biology, edaphology and geology, Faculty of Sciences (Biology), University of La Laguna, La Laguna, Santa Cruz De Tenerife Spain; 143 Forest Science, Kochi University, Monobe Otsu 200, Nankoku, Japan; 144 Juárez Autonomous University of Tabasco, Nanotechnology Engineering, Multidisciplinary Academic Division of Jalpa de Méndez, Carr. Estatal libre Villahermosa-Comalcalco, Km 27 S/N, C.P. 86205 Jalpa de Méndez, Tabasco, Mexico; 145 Unit Food & Agriculture, WWF-Netherlands, Driebergseweg 10, Zeist, The Netherlands; 146 Dpto. Ciencias, IS-FOOD, Universidad Pública de Navarra, Edificio Olivos - Campus Arrosadia, Pamplona, Spain; 147 Department of Soil, Water and Climate, University of Minnesota, 1991 Upper Buford Circle, St Paul, USA; 148 Earth Innovation Institute, 98 Battery Street Suite 250, San Francisco, USA; 149 University of California Davis, 1 Shields Avenue, Davis, USA; 150 Natural Resources & Environmental Management, University of Hawaii at Manoa, 1910 East West Rd, Honolulu, USA; 151 Natural Resource Sciences, McGill University, 21111 Lakeshore Road, Ste-Anne-de-Bellevue, Canada; 152 The Nature Conservancy, 4245 Fairfax Drive, Arlington, USA; 153 Animal Ecology, Justus Liebig University, Heinrich-Buff-Ring 26, Giessen, Germany; 154 Institute of Qinghai-Tibetan Plateau, Southwest Minzu University, Chengdu, China; 155 Laboratory of terrestrial ecosystems, Federal Research Centre “Kola Science Centre of the Russian Academy of Sciences”, Institute of North Industrial Ecology Problems (INEP KSC RAS), Akademgorodok, 14a, Apatity, Murmansk, Province Russia; 156 Key Laboratory of Geospatial Technology for the Middle and Lower Yellow River Regions (Henan University), Ministry of Education, College of Environment and Planning, Henan University, Kaifeng, China; 157 Faculty of Biological and Environmental Sciences, Post Office Box 65, FI 00014, University of Helsinki, Helsinki, Finland

**Keywords:** Biodiversity, Community ecology, Biogeography

## Abstract

Earthworms are an important soil taxon as ecosystem engineers, providing a variety of crucial ecosystem functions and services. Little is known about their diversity and distribution at large spatial scales, despite the availability of considerable amounts of local-scale data. Earthworm diversity data, obtained from the primary literature or provided directly by authors, were collated with information on site locations, including coordinates, habitat cover, and soil properties. Datasets were required, at a minimum, to include abundance or biomass of earthworms at a site. Where possible, site-level species lists were included, as well as the abundance and biomass of individual species and ecological groups. This global dataset contains 10,840 sites, with 184 species, from 60 countries and all continents except Antarctica. The data were obtained from 182 published articles, published between 1973 and 2017, and 17 unpublished datasets. Amalgamating data into a single global database will assist researchers in investigating and answering a wide variety of pressing questions, for example, jointly assessing aboveground and belowground biodiversity distributions and drivers of biodiversity change.

## Abstract (summary)

**Table:** 

Measurement(s)	earthworm communities • Abundance • organic material • Diversity • environmental properties
Technology Type(s)	digital curation
Factor Type(s)	location
Sample Characteristic - Organism	Lumbricina
Sample Characteristic - Environment	soil
Sample Characteristic - Location	global

Machine-accessible metadata file describing the reported data: 10.6084/m9.figshare.13399118

## Background & Summary

Soils are considered to be one of the most biodiverse terrestrial habitats^1–3^. Despite this, very little is known about the biodiversity that resides there compared to aboveground biodiversity, especially at the global scale^1,4,5^. This is surprising given the large number of local-scale biodiversity datasets available in the published literature. A number of studies have amalgamated local scale datasets, primarily for aboveground or marine organisms e.g.^6,7^, which can then be used for large-scale analyses e.g.^8,9^. Belowground biodiversity data are often overlooked in these large biodiversity databases^4^, and thus separate efforts to collate data are just now starting to emerge for certain belowground taxa, particularly microbes e.g.^10,11^.

Earthworms are involved in a large number of ecosystem functions and services, such as decomposition^12^, nutrient cycling^13^ and climate regulation^14^, amongst others^13^. In addition, they are often used as bioindicators of soil biodiversity and health^15^. Earthworms are relatively easy to sample; thus, a large amount of data are available^16^. Nevertheless, previous attempts to collate earthworm datasets have been geographically restricted^17,18^ or focused on country or regional species lists (e.g., DriloBASE; http://taxo.drilobase.org). By collating site-level diversity measures, we can also collect information on factors that might determine community composition, for example, measurements of soil properties or land use and cover.

**Fig. 1:**
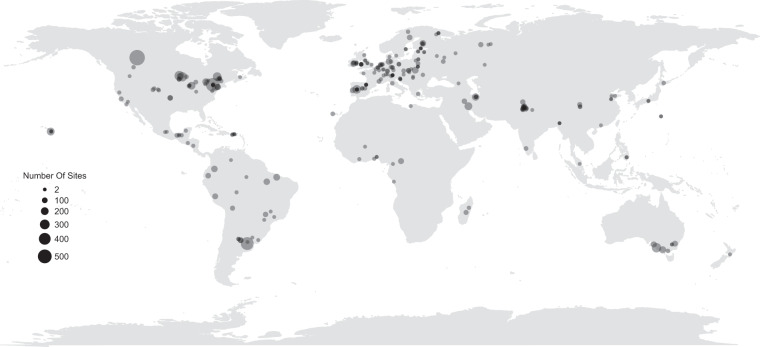
Locations of the 276 studies included in the database. Each circle represents the centre of a study (a collection of sites where earthworms were sampled with a consistent method). The size of the circle indicates the number of sites within the study. Transparency is used only for aiding visualisation.

Here, we describe a global database of local earthworm diversity and associated site-level characteristics from 10,840 sites in 60 countries (Fig. 1)^19^. Site-level information includes at least one sampled soil property, land use, and habitat cover for just over 58% of sites. Measurements of earthworm species richness (including species lists where available), total abundance, and biomass were collected at the site-level, and for some species occurrences i.e., abundance and biomass of the species recorded at a site. In addition, using expert opinion and details given by data providers, we classified each earthworm species into ecological groups based on their feeding and burrowing behaviours (epigeics, endogeics, anecics, epi-endogeics; more details below^20^).

The compilation of this dataset is timely. It can be used to answer long-standing questions in ecology in relation to this important belowground faunal group (e.g., global diversity patterns^16^). And in light of the IPBES Global Assessment^21^ and the loss of biodiversity, the dataset has the potential to be used to address the pressing issue of the consequences of environmental change on soil biodiversity. These data are suitable for linking with other soil databases, such as BETSI (http://betsi.cesab.org/), a database of soil organism traits^22^. Linking trait information with site-level diversity would then allow analyses of functional diversity. In addition, as nearly all sites have geographic coordinates, other environmental data layers (e.g., related to climate variables, land use or soil abiotic factors) could be linked to the site-level diversity measures (e.g.^16^,). Belowground diversity measures could also be linked to similar diversity measurements aboveground, thus enabling investigations across ecosystems to identify patterns of diversity and biodiversity changes^23^.

## Methods

This work was conceptualised and discussed during two ‘sWorm’ workshops in 2016 and 2017, funded by sDiv, the synthesis centre of the German Centre for Integrative Biodiversity Research (iDiv) Halle-Jena-Leipzig. More than 20 international scientists with expertise in earthworms, soil science, and/or data management met at each of the workshops.

On 18th December 2016, Web of Science was used to search the available literature for articles that had sampled the earthworm community. Keywords were used that captured measurements of diversity of all taxa within Oligochaetes: ((Earthworm* OR Oligochaeta OR Megadril* OR Haplotaxida OR Annelid* OR Lumbric* OR Clitellat* OR Acanthodrili* OR Ailoscoleci* OR Almid* OR Benhamiin* OR riodrilid* OR Diplocard* OR Enchytraeid* OR Eudrilid* OR Exxid* OR Glossoscolecid* OR Haplotaxid* OR Hormogastrid* OR Kynotid* OR Lutodrilid* OR Megascolecid* OR Microchaetid* OR Moniligastrid* OR Ocnerodrilid* OR Octochaet* OR Sparganophilid* OR Tumakid*) AND (Diversity OR “Species richness” OR “OTU” OR Abundance OR individual* OR Density OR “tax* richness” OR “Number” OR Richness OR Biomass))

This search returned 7,783 papers. All titles and abstracts of papers post-2000 were screened (6140 papers), and were excluded if they did not make reference to data suitable for the analysis. As it was most likely that raw data would need to be requested, papers in the literature search published before 2000 were not screened and excluded, as it was unlikely that available author contact details were up-to-date. After this initial screening, PDFs of all remaining papers (n = 986) were manually screened to determine whether data were suitable (see below). 477 papers made reference to data that was suitable.

In addition, to find unpublished data or to target underrepresented regions, inquiries were made to specific earthworm researchers regarding suitable datasets (e.g., by directly contacting researchers, giving presentations at the Second Global Soil Biodiversity Conference and the International Symposium of Earthworm Ecology). No date restrictions were placed on such datasets, and thus, some were published prior to 2000.

In order to be included in the database, the individual article was required to have sampled earthworm diversity using an appropriate quantitative methodology (such as hand-sorting of a soil quadrat e.g.^24^, or chemical expulsion e.g.^25^) at two or more sites that varied in their land-use/habitat cover or soil properties. At a minimum, we required data on the total abundance or fresh biomass of earthworms at each site, and if possible, the number of species (ideally with species binomials), and the abundance and biomass of each species. In addition, geographic coordinates of the sites were required, and at each site, data collectors ideally had sampled at least one of the following soil properties: soil pH (in H_2_O, KCl, CaCl_2_), soil organic carbon (%), soil organic matter (%), sand/silt/clay content (%), soil texture (USDA classification^26^), Cation Exchange Capacity (CEC), Base Saturation (%), Carbon:Nitrogen ratio, soil moisture (%), and soil type (WRB/FAO classification^27^).

Where possible, available data were extracted from the suitable articles. For each suitable article, the meta-data (e.g., the article title and DOI) was compiled (Online-only Table 1). Data were extracted from the article text, tables, figures, or supplementary material (e.g., using ImageJ^28^). Where data were not given but were required (Online-only Table 2), authors of the articles were contacted and the raw data (or missing information) were requested. If the authors did not respond, and the required information could not be obtained using an alternate method, the data were not entered into the database. All data were extracted into online data templates, with data from one article (i.e., a dataset) being entered into an individual template, referred to as a ‘file’. Each file was given a unique ID, and in total 199 files were created and made open-access.

A file could contain multiple ‘studies’, where each study was either a different sampling event i.e., multiple samples taken at the same site over time, and/or different sampling methodology. Each study was assigned a unique study ID. Sampled diversity of earthworms is highly dependent on the extraction method used^29^. If a dataset did not contain consistent sampling methodologies across all sites (i.e., some sites sampled with hand sorting and others hand sorting + chemical extraction), thus making it inappropriate to compare earthworm communities, the dataset was split into a separate study for each consistent methodology. If sites had been sampled multiple times, either across multiple years or within years, and the data were available for each sampling period, then only data from the first and the last sampling period were used. Each sampling period was entered as a study, which can help prevent temporal autocorrelation during analysis, e.g., when using a mixed-effects modelling approach.

A site was defined as a single location where the earthworm community was sampled using an appropriate quantitative methodology. Within each study, each site was given a unique ID (usually based on an ID given in the original source). For each site, information on the sampling methodology, soil properties, and land-use/habitat cover, along with the diversity measurements (site-level species richness, abundance and/or biomass) were entered into the data template (see Online-only Table 2 for full list of variables and the format that was required for the data template). Where possible, data were entered into the data template in the same format as given in the original source. To help enable this, columns often had separate fields to record the units. However, for some fields, values needed to be standardised prior to data entry, such as for the site coordinates and some soil properties (e.g., sand/silt/clay content).

All available and required soil properties for each site were entered into the template. Where a site had soil properties sampled at different depths (e.g., at 0–15, 15–30, and 30–40 cm), the weighted average of the values was entered into the templates. The value was then indicated as being a mean (Online-only Table 2).

**Fig. 2:**
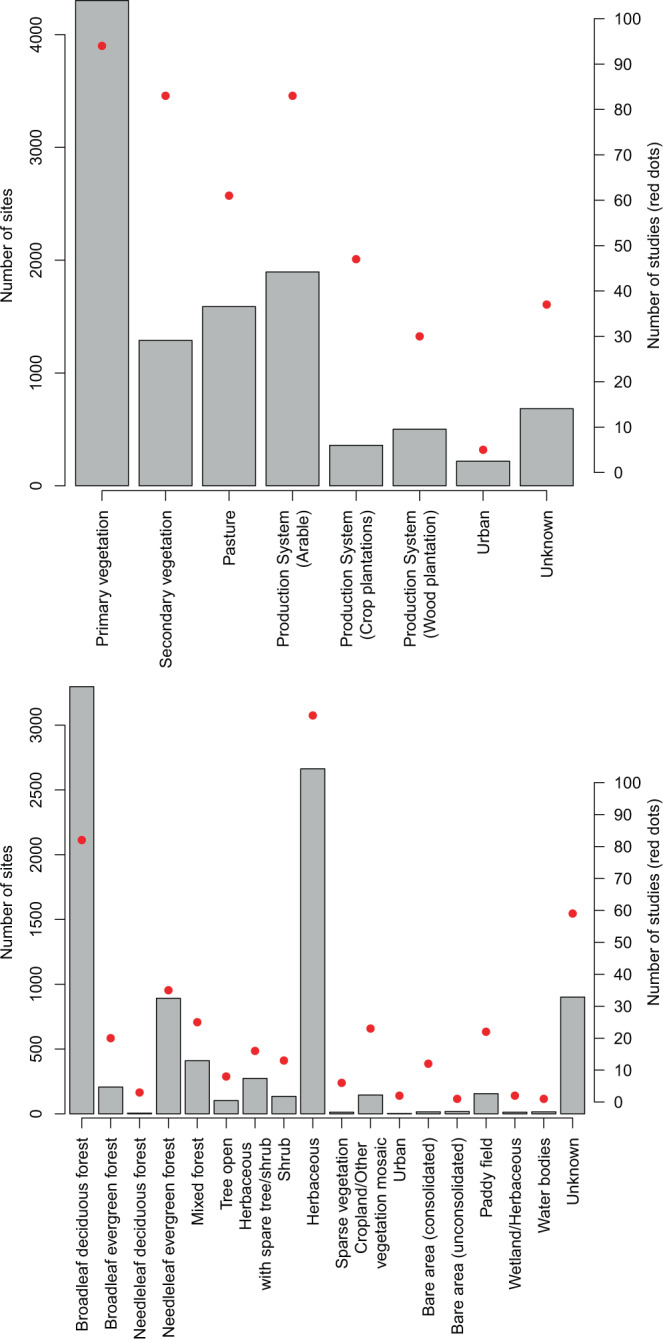
The number of sites (grey bars) and the number of studies (red dots) for each category in (a) the land-use system, and (b) the habitat-cover system. Sites could only be categorised within one category, but studies do contain sites that span multiple categories.

The fields for habitat cover, land-use, and management system were predefined categories based on ESA CCI-LC (https://www.esa-landcover-cci.org/), the Land-use Harmonization dataset^30,31^ (Fig. 2), and expert opinion (during the sWorm workshops), respectively. These classification systems were chosen based on knowledge of what external pressures might be important for explaining earthworm communities, whilst also ensuring consistency across all regions of the globe. Based on information given within the published article, or from the data providers directly, every site was classified into one of the categories for each of these fields. When information was missing, sites were classified as “unknown”. Additional information on the land use and management system classification definitions shown in Tables 1 and 2, respectively.

**Table 1:** Definitions for the land use category.

Land use category	Definition
**Primary**	Relatively undisturbed natural habitat
**Secondary**	Recovering, previously disturbed natural habitat
**Pasture**	Land used for the grazing of livestock
**Production - Arable**	Land used for crop production (e.g., wheat, rice, corn)
**Production - Plantations crops**	Land used for plantations crops (e.g., coffee, vineyards, oil palm)
**Production – Wood plantations**	Land used for timber production (e.g., teak)
**Urban**	Land converted to dense urban settlement
**Unknown**	If the land use is not given or is not clear

The land use classification was based on the Land-use Harmonization dataset^30,31^, to map to the original classification system, ‘Production – Wood plantations’ and ‘Production – Plantation crops’ would be ‘Secondary’ and ‘Production – Arable’ would be ‘Cropland’.

**Table 2:** A management classification system was created during the sWorm wokshops.

Management Intensity measure	Annual crops	Integrated systems	Perennial crops	Pastures (grazed lands)	Tree plantations
Tillage	×	×			
Pesticide	×	×	×	×	×
Fertilizer	×	×	×	×	×
Selectively harvested			×		×
Clear cut			×		×
Fire	×	×	×	×	×
Stocking rate				×	
Grazing all-year				×	
Rotation	×	×		×	
Monoculture	×	×	×	×	×
Planted				×	

For each managed site (i.e., not natural vegetation) the management system could also be identified (table headers), and additional management intensity variables could be also captured (table rows). However, not every management intensity variable was applicable for each management system, thus restrictions were placed. ‘×’ indicates which management intensity variable was applicable to each management system.

As sampling effort also impacts diversity measurements^32^, the sampling effort at each site was recorded. Effort was recorded in two ways:

1. The area that was sampled, e.g., of a quadrat or soil block, or the area across all e.g., quadrats. This depended on how the data were presented.
2. The number of times a site was sampled, either temporally or spatially. If a site was sampled over multiple time periods, it would be the number of occasions the site was sampled. If the site had multiple samples (e.g,, multiple quadrats) and the diversity measure is an average, the sampling effort would be 1. If the diversity is a total measure (e.g., the total number of species across all quadrats) the sampling effort would be the total number of e.g., quadrats.

When datasets contained information at a higher resolution than total abundance or biomass of earthworms at a site (i.e., at ecological group, genus, or species level), this information was entered into the species occurrence table (Online-only Table 3). Each row contained a measurement of an observation (e.g. species, morphospecies, genus, life stage or ecological group) at a single site. The measurement could be the presence only, abundance, or fresh biomass of the record. Where possible, for each row we also included the life stage (adult or juvenile), whether the species was native to the location or not, and the ecological group (epigeic, endogeic, anecic, epi-endogeic). Thus, if the diversity measure was for all the juveniles at the site regardless of species, columns such as the species binomial and genus would be empty, but life stage completed. Every species binomials and ecological group assignment were checked using DriloBASE and by earthworm taxonomists (GB, MJIB, MLCB, PL), see ‘Technical Validation’.

**Fig. 3:**
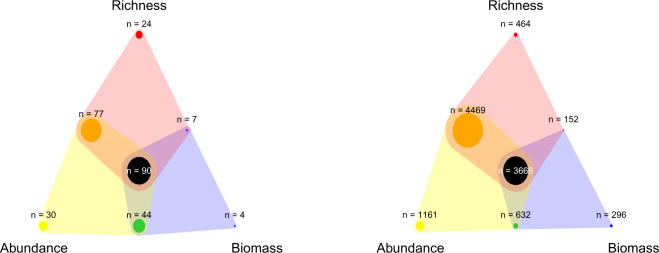
The number of (**a**) studies and (**b**) sites that measured each of the three community metrics. The points at the vertices indicate the number of studies or sites with only one community metric. The points on the edges indicates the number of studies or sites with the community metrics represented at the connecting two vertices. Finally, the point in the centre indicates the number of studies or sites with all three community metrics. For example, in (**a**), 145 studies measured biomass, shown in the blue polygon. 4 studies measured only biomass, 7 measured biomass and species richness, 44 measured biomass and abundance, and 90 measured all three metrics.

Where site-level diversity measures were given by the data provider, these were entered into the site-level sheet. Where site-level diversity measures were not given, but could be calculated from the species occurrence information, that was done in R^33^, following data entry and prior to subsequent analyses. The species present at each site, as given in the species occurrence data, were used for calculating species richness, this included species identified as sub-species. If data collectors identified a specimen as a morphospecies (i.e., a species delineation based solely on morphological characteristics, typically identified to genus level with a unique ID differentiating from other species of the same genus, as determined by the original data collector), it was included in the species richness estimate as an additional species. Unidentified species grouped as ‘unknown’ were excluded (Fig. 3). As juveniles of many earthworm species are hard to identify to species level^29,34^, juveniles were excluded from the calculation (even identified at family level). All earthworms (including juveniles) found at a site were included in the total biomass and abundance calculations.

After the ecological grouping (epigeic, endogeic, anecic, and epi-endogeic) of each species had been assigned and/or checked by the earthworm taxonomists, diversity measures within each ecological group at a site were also calculated. As with the site-level metrics, the species richness within each ecological group was calculated using only species with binomials or morphospecies. Biomass and abundance of each ecological group at a site was calculated regardless of species identity. The total number of the ecological groups at each site was calculated regardless of abundance, biomass, life stage or native status of the species included (maximum ecological group richness = 4).

## Data Records

The data presented here are available in the iDiv data portal (10.25829/idiv.1880-17-3189. Dataset ID: 1880)^19^ in a static form. In addition, the full dataset will be hosted by Edaphobase (www.portal.edaphobase). In the future, the version in Edaphobase might change (i.e., with species names revisions, or requests from the data providers) and will hopefully be added to with additional earthworm records (or other soil taxa).

The data is stored in three tables; meta-data (Online-only Table 1), site-level (Online-only Table 2), and species occurrence (Online-only Table 3). The file ID links the meta-data to the site-level data, and the Study ID and the Site ID, link the site-level data to the species occurrence table.

For all suitable datasets, the meta-data information was completed. The meta-data contains bibliographic information on the original paper which analysed, or published, the data, as well as contact information of the person who provided the raw data (not included in the release of the database for privacy reasons). The meta-data also included the number of sites and studies within the file, so that validation checks could be completed. Online-only Table 1 shows all fields within the meta-data, personal information of data providers has not been made available.

Information on all sampled sites within each dataset was recorded in the site-level table (Online-only Table 2). Each row represents a single site within a study, with information on the sampling methodology, soil properties, and how the land was used, managed, and covered. The site-level earthworm community metrics (species richness, abundance and biomass) are also included if available.

Site-level species lists, or abundance, and/or biomass measures for individual records are given in the species occurrence table (Online-only Table 1). Each row is a measurement of an observation at a site (22,690 non-zero observations in total). An observation could relate to a species (with a scientific binomial, e.g., the abundance of *Lumbricus terrestris* at a site, or a morphospecies identification), a genus, life stage, ecological group, or native/non-native group (e.g., the abundance of all non-native species at a site). Details of native/non-native status of a species was only available when provided by the original data collector.

## Technical Validation

Templates used to enter the individual datasets were designed so that fields were only allowed certain values and formats where possible. This helped to reduce spelling errors, slight inconsistencies, and incorrect values being entered. Data providers were contacted if details within their raw data were unclear. As multiple people entered data into the templates, detailed documentation was created at the start of the project to ensure consistency amongst those involved. In addition, a subset of datasets was checked by several curators.

All earthworm species names were checked against DriloBASE (http://taxo.drilobase.org) to identify potential synonyms and spelling mistakes. Following that, earthworm specialists and taxonomists (GB, MJIB, MLCB and PL) checked the scientific names, removed synonyms and updated names if taxonomies had changed. Where ecological groupings were missing, the earthworm taxonomists also added them where possible, based on the available literature.

## Usage Notes

Land-use fields were based on classification schemes, and may not be the most suitable for the analysis of earthworms. We included a free-text field (“Habitat as described”) that could be used by future researchers to define their own classification scheme for land-use or habitat cover.

**Fig. 4:**
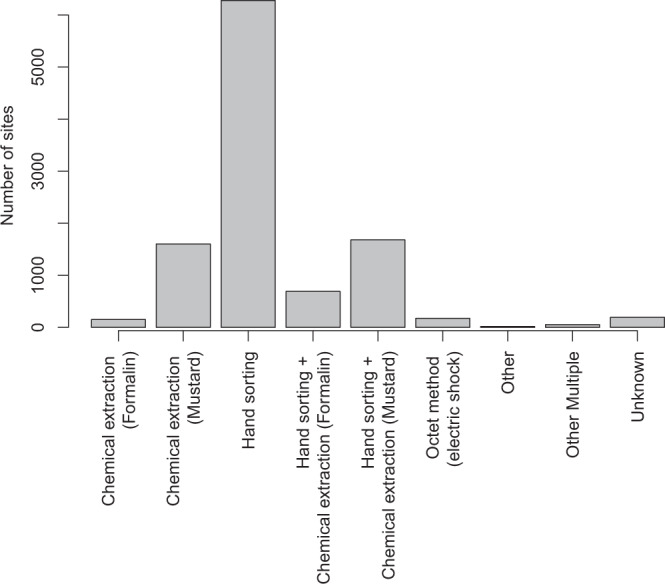
The number of sites sampled with each sampling method across the different earthworm studies.

As diversity measures are highly influenced by sampling methodology, we included information on sampling methods in the database (Fig. 4). In addition, we would expect that variation in diversity would differ between the individual datasets due to, for example, inter-observer variability. We highly recommend that statistical methods used on this database take these between-dataset variations into account.

Despite our efforts to obtain a global dataset, there is a geographic bias (Fig. 1), such that sites are highly clustered in certain regions (e.g., Europe), sparse in others (e.g., South America), or lacking (e.g., southern Africa, northern Russia). To reduce such biases, we attempted to contact as many researchers as possible in such areas to acquire data. Although this helped to improve the data coverage, it did not remove the gaps. We hope to address these gaps in the future, but in the meantime, researchers should be aware of the influence these biases might have on their analyses^35,36^.

## Data Availability

All code used to format and clean the dataset for publication is available on GitHub (www.github.com/helenphillips).

## Online-only Tables

**Online-only Table 1:** Information captured in the data template relating to meta-data.

Field	Format	Information	Required field (*)
File	Text	Unique ID for each article	*
Article_Title	Text	Title of the article the data was published in. If unpublished then NA or “Unpublished”	
Article_Year	Integer	The year of the article was published (NA if unpublished)	
Article_FirstAuthorSurname	Text	The surname of the first author of the article	*
PaperContact_Surname	Text	The surname of the corresponding author of the article	
Article_Journal	Text	The journal the article was published in (NA if unpublished)	
Article_DOI	Text	The DOI of the article (NA if not available or unpublished)	
Data_DOI	Text	The DOI of the data (if different from the article, NA if not available)	
Number_of_Studies	Integer	The total number of studies in the file	*
Total_Number_ofSites	Integer	The total number of sites across all studies in the file	*
Total_Number_ofSpecies	Integer	The total number of species in the dataset (if given)	
Entire.Community	Yes/no	Was the entire earthworm community sampled, or just selected species. If this information is unknown or unclear NA is given	*
Data.From.Paper	Yes/no	Was the data taken from the paper, or did the author provide raw values	*
Other.soil.organisms.sampled	Yes/no	Were any other soil organisms sampled at the same sites	*

**Online-only Table 2:** Information captured in the data template, relating to site-level information.

Field	Format	Possible Values	Information	Required field
File	Text		Unique ID for each article. Assigned to all studies within a single publication	*
Study_Name	Text		Unique ID for each study. Assigned to all sites within a single study	*
Site_Name	Text		Unique ID given to each site. A site that is sampled in different studies will have the same ID	*
Observational	Multiple choice	Observation; Experimental	Was the data from an observational study or an experimental study. Experimental studies may be unrealistic in their treatments, and are often over a smaller area (resulting in similar/identical coordinates)	*
Latitude (decimal degrees)	Numerical		The latitude of the site (in decimal degrees only)	*
Longitude (decimal degrees)	Numerical		The longitude of the site (in decimal degrees only)	
Altitude (m)	Numerical		The altitude of the site (in metres only)	
Country	Text		The country the site was in (as given by data collector)	*
Sample_StartDate_Month	Integer	1–12	The month the sampling started	
Sample_StartDate_Year	Integer	Less than 2018	The year the sampling started	*
Sample_EndDate_Month	Integer	1–12	The month the sampling ended	
Sample_EndDate_Year	Integer	Less than 2018	The year the sampling ended	
ExtractionMethod	Multiple choice	Visual search, Hand sorting, Chemical extraction (Mustard), Chemical extraction (Formalin), Octet Method (electric shock), Hand sorting + Chemical extraction (Mustard), Hand sorting + Chemical Extraction (Formalin), Other, Other Multiple, Unknown	The methodology used to sample the earthworms	*
Sampled Area	Numerical		The area over which sample(s) were taken. Typically the size of the quadrat or soil block	*
Sampled Area Unit	Multiple choice	cm^2^, cm^3^, m^2^, m^3^, Unknown, Other	The unit of the sampled area	*
Sampling Effort	Numerical		The number of times the site was sampled to obtain the earthworm community metric(s) provided	*
pH	Numerical		Sampled pH of the site	At least one required
pH Collection Method	Multiple choice	H_2_O, KCl, CaCl_2_, Other, Unknown	The suspension solvent used in the measurement	
pH_mean	Multiple choice	Yes/no	Denotes whether the value given for the pH is a mean from individual values across a depth profile	
CEC	Numerical		The Cation Exchange Capacity of the site	
CEC_unit	Text		The unit of the CEC measurement	
XXX_mean	Multiple choice	Yes/no	Denotes whether the value given for the pH is a mean from individual values across a depth profile	
Base Saturation(%)	Numerical		The base saturation of the site	
BaseSaturation_mean	Multiple choice	Yes/no	Denotes whether the value given for the base saturation is a mean from individual values across a depth profile	
Organic Carbon (%)	Numerical		The organic carbon content of the soil at the site	
OC_mean	Multiple choice	Yes/no	Denotes whether the value given for the organic carbon is a mean from individual values across a depth profile	
Soil Organic Matter (%)	Numerical		The soil organic matter content at the site	
SOM_mean	Multiple choice	Yes/no	Denotes whether the value given for the soil organic matter is a mean from individual values across a depth profile	
C/N ratio	Numerical		The carbon to nitrogen ratio at the site	
CN_mean	Multiple choice	Yes/no	Denotes whether the value given for the carbon:nitrogen ratio is a mean from individual values across a depth profile	
Sand (%)/Silt (%)/Clay (%)	Numerical		The percentage of sand, silt and clay at the site	
Sand_silt_clay_mean	Multiple choice	Yes/no	Denotes whether the value given for the percentage of sand, silt and clay is a mean from individual values across a depth profile	
USDA_SoilTexture	Multiple choice	clay, sandy clay, sandy clay loam, clay loam, loam, sandy loam, loamy sand, sand, silt clay, silty clay loam, silt loam, silt	The texture of the soil at the site	
Soil Moisture(%)	Numerical		The soil moisture at the site	
WRB/FAO_SoilType	Multiple choice	Acrisols, Albeluvisols, Alisols, Andosols, Anthrosols, Arenosols, Calcisols, Cambisols, Chernozem, Cryosols, Durisol, Ferralsols, Fluvisol, Gleysols, Gypsisols, Histosols, Kastanozem, Leptosols, Lixisols, Luvisols, Nitisols, Phaeozem, Planosols, Plinthosols, Podzols, Regosols, Retisols, Solonchaks, Solonetz, Technosols, Umbrisols, Vertisols	The type of soil at the site. Using the WRB/FAO classification^26^, but only classified when given by the data providers in the same system	
LandUse	Multiple choice	Primary vegetation, Secondary vegetation, Production - Arable, Production - Crop plantations, Production - Wood plantation, Pasture, Urban, Unknown	The category of land use^29,30^ that the site was classified as. Classification was based on descriptions of the site in the text of the original publication or subsequent correspondence with the data provider	*
HabitatCover	Multiple choice	Broadleaf evergreen forest, Broadleaf deciduous forest, Needleleaf evergreen forest, Needleleaf deciduous forest, Mixed forest, Tree open, Shrub, Herbaceous, Herbaceous with spare tree/shrub, Sparse vegetation, Cropland, Paddy field, Cropland/Other vegetation mosaic, Mangrove, Wetland, Bare area (consolidated, e.g. rock), Bare area (unconsolidated, e.g. sand), Urban, Snow/Ice, Water bodies, Unknown	The category of habitat cover (ESA CCI-LC 300 m; https://www.esa-landcover-cci.org/) that the site was classified as. Classification was based on descriptions of the site in the text of the original publication or subsequent correspondence with the data provider	*
Management System	Multiple choice	Annual crop, Integrated systems, Perennial crops, Pastures (grazed lands), Tree plantations, Unknown, NA	The management system at the site. Sites with no management, i.e. pristine or recovering sites, were categorised as ‘NA’. Classification system based on expert opinion.	
Tillage/Pesticide/Fertilizer/Selectively harvested/Clear cut/Fire/Grazing all year/Rotation/Monoculture/Planted	Boolean		The presence or absence of each pressure at the site. The applicability of these depended on the ‘management system’. Thus, they were left empty or filled as ‘NA’ when not applicable (see Supplementary Material 1)	
Habitat as described	Text		Free text field for a description of the site based on the original article or on emails from the data provider	*
SpeciesRichness	Numerical		The species richness at the site (if available)	At least one required
SpeciesRichnessUnit	Multiple choice	Number of species, Species per cm^2^, Species per cm^3^, Species per m^2^, Species per m^3^, Other	The units of the species richness value	
Site_WetBiomass	Numerical		The total wet biomass of the site (if available)	
Site_WetBiomassUnits	Multiple choice	g, g/m^2^	The units of the biomass value	
Site_Abundance	Numerical		The total abundance of the site (if available)	
Site_Abundance Units	Multiple choice	Number of individuals, Individuals per cm^2^, Individuals per cm^3^, Individuals per m^2^, Individuals per m^3^	The units of the abundance value	

**Online-only Table 3:** The information captured in the “species occurrence” data sheet. An observation could refer to a species (either with a scientific binomial or a morphospecies identification), or a genus, life stage, ecological group, or native/non-native group.

Field	Format	Possible Values	Information	Required field
File	Text		Unique ID for each article. Assigned to all studies within a single publication	*
Study_Name	Text		Unique ID for each study. Assigned to all sites within a single study	*
Site_Name	Text		Unique ID given to each site. A site that is sampled in different studies will have the same ID.	*
OriginalSpeciesBinomial	Text		The species binomial of the observation as given by the data collector, prior to revision by earthworm experts	
SpeciesBinomial	Text		The species binomial of the observation (following revision by earthworm experts)	At least one required
MorphospeciesID	Text		An indicator (i.e., number or letter) of a observation that has been only identified to morphospecies	
Genus	Text		The genus of the observation	
Family	Text		The family of the observation	
Ecological_group	Multiple choice	Epigeic, Anecic, Endogeic, Epi-Endogeic, Unknown	The ecological group of the observation (following revision by earthworm experts)	
LifeStage	Multiple choice	Adult, Juvenile, Unknown	The life stage of the observation	
Native/Non-native	Multiple choice	Native, Non-native, Unknown	Whether the observation is native or non-native in the sampled region	
Abundance	Numerical		The total abundance of the observation at a specific site	
Abundance Unit	Multiple choice	Number of individuals, Individuals per cm^2^, Individuals per cm^3^, Individuals per m^2^, Individuals per m^3^	The units of the abundance value	
WetBiomass	Numerical		The total biomass of the observation at a specific site	
WetBiomassUnits	Multiple choice	g, g/m^2^	The units of the biomass value	

For each dataset, this datasheet was only used if species occurrence data were available.
